# A psychometric assessment of a novel scale for evaluating vaccination attitudes amidst a major public health crisis

**DOI:** 10.1038/s41598-024-61028-z

**Published:** 2024-05-04

**Authors:** Linan Cheng, Jianhui Kong, Xiaofeng Xie, Fengying Zhang

**Affiliations:** 1 School of Nursing, Soochow University, Suzhou, Jiangsu China; 2 West China Hospital/West China School of Nursing, Sichuan University, No.37, Guoxue Alley, Chengdu, 610041 China; 3 Law School of Southwest Minzu University, Chengdu, Sichuan China; 4 Innovation Center of Nursing Research, Nursing Key Laboratory of Sichuan Province, West China Hospital, Chengdu, Sichuan China

**Keywords:** Acceptance attitudes, Immunization, Vaccination attitudes, Public health crisis, Health care, Medical research, Risk factors

## Abstract

Despite abundant scientific evidence supporting immunization benefits, vaccine hesitancy remains a significant global health concern, particularly during public health crises. Exploring public attitudes towards vaccination is crucial. This study aimed to develop and validate a tailored Public Vaccination Attitudes Scale specifically under the unique circumstances of a public health crisis. A psychometric evaluation was conducted using a cross-sectional study during the peak of a major public health crisis. The scale was developed and its psychometric properties validated using three approaches: (1) generating the item pool through literature research and focus group discussions; (2) assessing the items through expert consultation; and (3) evaluating construct validity, content validity, and internal consistency reliability through exploratory factor analysis (EFA) and confirmatory factor analysis (CFA). Data from a total of 3921 respondents were randomly divided into two subsets, one for EFA (n = 1935) and the other for CFA (n = 1986). A 22-item draft scale with five factors was created after literature research and focus group discussion. The content validity of this scale ranged between 0.88 and 1.00. EFA showed a 17-item scale with four factors (Cronbach’s α > 0.7) accounting for 68.044% of the total variance. CFA showed that the values of the fit indices, including convergent validity and discriminant validity, were excellent or acceptable. The overall Cronbach’s α was 0.874, and each factor ranged from 0.726 to 0.885. This study introduces a valuable tool for assessing vaccination attitudes during public health crises, aiding researchers, policymakers, and nurses in combating vaccine hesitancy. Emphasizing the importance of fostering vaccine acceptance, it enhances disease control during emergencies, contributing to the knowledge needed for more effective public health strategies and crisis responses

## Introduction

Public health crises, often characterized by their sudden and unpredictable nature, exert a profound impact on societies and global healthcare systems. These crises can be triggered by various factors, including infectious disease outbreaks, natural disasters, or unforeseen events. During such crises, the public’s stance on vaccination assumes a pivotal role in the management and control of diseases^1^.

Immunization stands as one of the most successful and cost-effective health interventions against infectious diseases, particularly in the context of epidemic disease prevention and control^2^. Research has shown the significant advantages of vaccination, encompassing decreased disability, hospital admissions, healthcare costs, and mortality rates^3–5^. However, recent years have witnessed a concerning decline in public trust in vaccines, accompanied by rising rates of vaccine hesitancy^6,7^. Vaccine hesitancy, shown as reluctance or refusal to get vaccinated despite vaccines being available, is an intriguing phenomenon, especially during crises such as the COVID-19 pandemic. It has gained more attention because it can weaken public health efforts^8^. Even before the onset of the COVID-19 pandemic, the World Health Organization (WHO) ranked hesitation about vaccines as one of the top ten global health threats^9,10^.

Understanding global drivers of vaccine acceptance is a pressing concern^11^. Vaccine acceptance, a complex interplay of individual characteristics, social environment, and cross-cultural differences^4,12^, impacts disease risk perception, vaccine attitudes, and overall demand among the population. High vaccination coverage is crucial, especially during outbreaks of emerging infectious diseases^13^. Misconceptions about vaccine safety and disease risks will lead to hesitancy or refusal^14^. Comprehending the intricate interplay of factors impacting vaccine acceptance aids in making well-informed decisions for public health practitioners, policymakers, and researchers.

In 2017, Martin and Petrie developed the Vaccination Attitudes Examination Scale (VAX) through literature review and focus group data analysis^15^. It’s a concise and comprehensive tool focused on identifying individuals with vaccination reluctance, targeting four key factors: mistrust of vaccine benefits, concerns about unforeseen future, worries about commercial profiteering, and preference for natural immunity. The VAX has undergone rigorous testing of its psychometric properties in several countries, including Hebrew^4^, Spanish^16^, French^17^, Colombian^18^, and Romanian^19^. And the VAX has also been adapted to specially assess attitudes towards vaccines against COVID-19^4,20–22^. And the VAX has consistently shown good reliability and validity. However, notable fluctuations in vaccine acceptance rates have been observed, especially during the COVID-19 period.

Significant changes in vaccination attitudes have been observed across the pandemic.98.7% of participants received at least one dose of a COVID-19 vaccine^23^, with 9% refusing vaccination and 30.8% expressing hesitancy^24^. Studies have also shown that 46.6% of participants displayed a positive attitude towards COVID-19 vaccination uptake^25^, while 45.6% were hesitant about COVID-19 vaccines^22^. Concerns regarding unexpected future effects have increased, indicating higher perceived risks related to vaccination, and a more deliberative information-seeking approach among participants^26^. Vaccine acceptance attitudes are influenced by sociodemographic factors like gender, age, occupation, past vaccination success, and education level^23–26^, which are linked to participants' perception of disease risk. When perceiving low disease risk, individuals may hesitate to accept vaccination risks^27^.

Significant fluctuations in vaccination attitudes highlight the limitations of VAX and other related measurement tools during major public health events. Attitudes toward vaccination are influenced by situational factors, particularly during uncertain Major Public Health Crisis events. The balance between individuals' risk perception of the event and the perceived safety of vaccines primarily determines vaccine acceptance attitudes. Although the adjusted VAX assessed acceptance attitudes and influencing factors during COVID-19, the focus was primarily on vaccine safety, neglecting individuals’ risk perception of the crisis event itself. When personal safety is not directly threatened, people tend to prioritize risk avoidance over vaccine acceptance^1^. Therefore, integrating risk perception factors into the scale is necessary. Additionally, vaccination attitudes are influenced by multicultural factors. Despite cultural adjustments in numerous studies, the accuracy of measurement depends on whether the content reflects the genuine needs of specific cultural populations. Thus, integrating specific cultural contexts is necessary to establish reliable measurement indicators. Furthermore, during a Major Public Health Crisis, rapid and decisive response measures are essential. Measurement tools must have concise and rapid identification capabilities, as well as universality across different populations. Given the differences in vaccine acceptance attitudes across various countries and cultural backgrounds. Thus, the purpose of this study is to develop the Public Vaccination Attitudes Scale to assess and measure public attitudes towards vaccination during a major public health crisis.

This study contribute to addressing the critical gap in our understanding of vaccination attitudes amidst public health crises and provide a validated instrument for evaluating these attitudes. By doing so, it seeks to contribute to the development of more effective strategies for vaccine acceptance, ultimately improving disease control and response efforts during times of crisis.

### Scale development

Three approaches were employed in developing the Vaccination Attitudes Amidst a Major Public Health Crisis and determining its psychometric properties^3^: (1) generation of an item pool; (2) expert consultation; and (3) factor analysis.

### Generation of an item pool

Content analysis of the literature and focus group discussion were conducted at this stage^24^. The items of the scale were obtained by reviewing the literature on measurement tools or questionnaires that could be related to vaccination and conducting focus group discussions covering topics such as factors that affect acceptance or refusal of vaccination and concerns about vaccination. A 22-item draft scale was created with 5 factors, recognition, environment, value, security and knowledge, with higher scores representing positive acceptance attitudes. Responses were provided on a 5-point scale (1 = strongly disagree to 5 = strongly agree). Items 4, 5, 6, 8, 9, 12, 13, 14 and 20 were scored in the opposite order. The overall score ranged from 22 to 110 points.

### Expert consultation

Item evaluation was performed by the expert consultation method. Inclusion criteria were as follows: ① Expertise in hospital management, disaster management, public health management, vaccine research, and participation in public health prevention and control; ② greater than 5 years of professional experience; ③ bachelor’s degree, associate senior title, or above; and ④ voluntary participation. Experts who could not be contacted electronically were excluded. A draft scale was sent to 20 experts at this stage, with 18 experts responding. If no response was received within 2 weeks, we considered it a lack of time or interest and discontinued contact. The experts classified each item of the scale as ‘unimportant', ‘important but needs correction', and ‘important'.

Expert Information: ① Age ranged from 30 to 60 years old, with one expert above 60 years old.; ② 13 (72.2%) had 31–40 years of experience, 2 (11.1%) had 21–30 years, 2 (11.1%) had 11–20 years, and only 1 (5.6%) had 5–10 years; ③ 3 (16.7%) had undergraduate degrees, 4 (22.2%) had master’s degrees, and 11 (61.1%) had doctoral degrees.; ④ 4 (22.2%) were head nurses in hospitals, 4 (22.2%) were hospital managers, 2 (11.1%) worked in public health management, 2 (11.1%) were involved in vaccine research, 5 (27.8%) were engaged in combating a public health crises, and 1 (5.6%) was a professor of statistics.; ⑤ the research team comprised 1 PhD student and three master’s students. In both rounds of expert consultation, the judgment coefficient (Ca) was 0.79, the familiarity coefficient (Cs) was 0.87 and 0.95 respectively, and the expert authority coefficient (Cr) was 0.83 and 0.87. A Cr value ≥ 0.7 is generally considered acceptable for reliability^28^. The consulted experts demonstrated a high level of authority on the issues, ensuring highly reliable results.

The recovery rate, content validity index (CVI), item content validity index (I-CVI), scale-level S-CVI, average S-CVI (S-CVI/Ave) and universal agreement S-CVI (S-CVI/UA) were calculated.

The consensus coefficients for the two rounds were 90% and 93.3% respectively. All I-CVI data were greater than 0.88^28^. The experts' active participation and efficiency in both rounds demonstrate their keen interest in the study and reflect heightened global awareness and concern stemming from significant public health events like COVID-19. Vaccine acceptance plays a crucial role in safeguarding global health, thus making research on vaccination attitudes a prominent focus and trend.

In the first round of expert consultation, one suggestion was added: “Since the vaccine is free, I’m willing to take it.” Additionally, five recommendations were modified. No items were added to or removed from the scale, but eleven items underwent corrections to rectify grammatical and spelling errors and enhance statement clarity. After the modifications, Kendall’s W coefficient was 0.136 (*P* < 0.05), S-CVI/UA was 0.66, and S-CVI/Ave was 0.97. Following these revisions, we proceeded with the second round of expert consultation. The Kendall’s W coefficient for the second round of Delphi consultation was 0.171 (*P* < 0.001). S-CVI/UA was 0.87, and S-CVI/Ave was 0.99. The 22-item scale was created according to expert suggestions. All indicators showed that the scale exhibited relatively satisfactory content validity^29^ and was recognized by experts from a professional point of view.

### Factor analysis

EFA and CFA were performed to determine the construct validity together with the reliability of the scale, constituting the psychometric evaluation.

Item analysis and homogeneity tests were used to filter the initial items. This study adopted the extreme group method to analyse items. The difference between the top 27% and the bottom 27% of the total score of the questionnaire was compared by t tests. If *P* > 0.05, the item was deleted. We compared the high group (top 27%, total score ≥ 155 points) with the low group (bottom 27%, total score ≤ 82 points), and all items achieved significant P values (*P* < 0.001). In the homogeneity test, the correlation coefficient (R value) between items and the whole questionnaire was higher than 0.4, indicating that the acceptance attitudes measurement tool developed in this study exhibited a high level of homogeneity.

Kaiser–Meyer–Olkin (KMO) and Bartlett’s Test of Sphericity were performed to assess the suitability of the questionnaire for exploratory factor analysis. A KMO value > 0.80 or a *P* value of Bartlett’s Test < 0.05 indicates adequate sample size and suitability of variables for factor analysis. A further factor structure was developed with factors whose Cronbach’s α was > 0.7 and item loading was > 0.4, which were then selected as the new structure. Confirmatory factor analysis (CFA) was used to test whether this factor structure was suitable, and convergent validity and discriminant validity were explored. Cronbach’s α coefficient was used to assess the reliability of the scale.

## Methods

### Study design

The research adopted a cross-sectional design and adhered to the STROBE cross-sectional reporting guidelines^25^. Participation in the study was voluntary and anonymous, with informed consent obtained and documented via electronic signature. All collected data were treated confidentially and utilized solely for research purposes.

### Data collection

Data collection occurred from June to August 2021, facilitated by a Sojump online survey, accessible through the Questionnaire Star survey website, the largest online survey platform in China, catering to research institutions' online questionnaire design and survey needs. Employing the self-selection online survey method, a nonprobability sampling approach^26^, participants were recruited via social network links. The study’s target population comprised adults aged 18 years or older residing in mainland China.

### Quality control

Throughout the investigation, two researchers were tasked with overseeing the completion of questionnaires and gathering adjustment recommendations via the website. Subsequently, upon concluding the investigation, we meticulously screened the data to ensure its accuracy.

### Floor and ceiling effects

Among the participants, 8% scored the lowest possible score for the survey, and 5% scored the highest possible score. We introduced reverse items to address ceiling or floor effects. Additionally, the diverse participant pool and random data division into two parts for exploratory and confirmatory factor analyses also helped alleviate these effects to some extent.

### Participants

A total of 4021 respondents participated in the survey using the Questionnaire Star survey website. However, 100/4021 (2.5%) of the questionnaires were removed from the sample according to the following exclusion criteria: (a) the surveys were incomplete; (b) the questionnaires were completed by respondents under 18 years of age; (c) the respondents did not consent using the first two questions; and (d) the same response options were chosen consistently. Ultimately, 3921 eligible surveys were analysed.

### Statistical analysis

Data were analysed by IBM SPSS 20.0 for Windows and AMOS, and *P* < 0.05 was considered statistically significant in two-tailed tests. The general characteristics were described by means (SD) or frequencies for categorical items. Concordance among the experts was assessed using Kendall’s W analysis. The structural validity of the scale was assessed using EFA and CFA. Cronbach’s alpha was used for reliability analysis.

### Ethics approval and consent to participate

The West China Hospital of Sichuan University Biomedical Research Ethics Committee approved the study (ethics number: 2021-992). All participants gave electronic informed consent for the participation. The authors assert that all procedures contributing to this work comply with the ethical standards of the relevant national and institutional committees on human experimentation and the Helsinki Declaration of 1975, as revised in 2008. Participation in the study was anonymous.

## Results

### Demographic characteristics

The study ensured the representativeness of survey participants by encompassing all adults aged 18 years and above nationwide. Participants had a mean age of 32.58 years (SD = 11.85; range = 18–85). Among the respondents, 77.5% were female (n = 3073), and 94.8% were ethnically Han (n = 3716). The highest educational level of 28.1% was a junior college degree (n = 1100). Almost half were married (51.3%). A total of 75.8% had a nonmedical background (2971), and 50.1% reported that their families had a medical background (1966). A total of 31.9% came from a large city, and 31.5% were from a mid-sized city. A total of 29.6% had an average family income (CNY) ≤ 5000 (Table 1).

**Table 1:** Characteristics of the participants.

Variables		N (%)	Variables		N (%)
Age group	18–25	1481 (37.8)	Average family income(CNY)	≤ 5000	1162 (29.6)
	26–30	543 (13.8)		5001–8000	945 (24.1)
	31–40	870 (22.2)		8001–10,000	621 (15.8)
	41–50	708 (18.1)		10,000–20,000	774 (19.7)
	51 and above	319 (8.1)		> 20,000	419 (10.7)
Gender	Female	3037 (77.5)	Family medical background	Yes	1966 (50.1)
	Male	884 (22.5)		No	1955 (49.9)
Highest level of education	Senior high school and below	982 (25.0)	Minority	Han	3716 (94.8)
	Junior college	1100 (28.1)		Other	205 (5.2)
	Bachelor	1090 (27.8)	Areas	Eastern China	689 (17.6)
	Master and above	749 (19.1)		South China	428 (10.9)
Permanent living region	Big city	1251 (31.9)		Central China	371 (9.5)
	Middle city	1234 (31.5)		North China	561 (14.3)
	County	880 (22.4)		Northwest China	341 (8.7)
	Rural	556 (14.2)		Southwest China	1424 (36.3)
Marriage status	Married	2011 (51.3)		Northeast China	107 (2.7)
	Unmarried	1802 (46.0)	Professional background	Medical background	1500 (38.3)
	Others (divorced or widowed)	108 (2.8)		Non-Medical background	2421 (61.7)

### Item analysis and item homogeneity test

The questionnaire was randomly divided into two parts, one for EFA (n = 1935) and the other for CFA (n = 1986). The results of item analysis and item homogeneity were based on EFA. All items achieved significant *P* values (*P* < 0.001). In the homogeneity test, the correlation coefficient (*R* value) between items and the whole questionnaire was higher than 0.4; the value for Item 10 was 0.395, so it was deleted. The remaining 21 items were included in the EFA (Table 2).

**Table 2:** Item analysis and item homogeneity test.

Item	t	*P*	CI(95% )	R
A1	− 24.371	0.000	− 1.118 to −0 .951	0.623**
A2	− 24.790	0.000	− 1.108 to 0.945	0.605**
A3	− 24.724	0.000	− 1.069 to 0.912	0.590**
A4	− 24.379	0.000	− 1.656 to − 1.409	0.536**
A5	− 28.715	0.000	− 1.418 to − 1.237	0.621**
A6	− 28.498	0.000	− 1.425 to − 1.241	0.631**
A7	− 21.334	0.000	− 1.203 to − 1.000	0.490**
A8	− 29.563	0.000	− 1.596 to − 1.397	0.632**
A9	− 24.035	0.000	− 1.455 to − 1.235	0.527**
A10	− 15.030	0.000	− .912 to − 0.701	0.395**
A11	− 23.280	0.000	− 1.034 to − 0.874	0.550**
A12	− 23.164	0.000	− 1.531 to − 1.292	0.523**
A13	− 22.914	0.000	− 1.535 to − 1.293	0.466**
A14	− 28.962	0.000	− 1.491 to − 1.302	0.603**
A15	− 31.829	0.000	− 1.422 to − 1.256	0.682**
A16	− 31.951	0.000	− 1.535 to − 1.358	0.662**
A17	− 31.898	0.000	− 1.397 to − 1.235	0.692**
A18	− 28.182	0.000	− 1.424 to − 1.239	0.635**
A19	− 16.501	0.000	− 1.224 to − 0.964	0.426**
A20	− 17.744	0.000	− 1.317 to − 1.055	0.412**
A21	− 33.563	0.000	− 1.229 to − 1.093	0.713**
A22	− 29.991	0.000	− 1.090 to − 0.956	0.619**

**P < 0.01.

### Structural validity and reliability

Principal component analysis and the promax method were used in EFA. Factors with eigenvalues above one were selected, while items with a maximum factor loading value < 0.4 and those with double loading were deleted if the factor load value of the main item was at least 2 times that of the minor factor as the entry inclusion criterion. The KMO value was 0.888, and Bartlett’s test of sphericity was significant (X^2^ = 17999.208, df = 136, *P* < 0.001), the data were ideal for factor analysis. The cumulative contribution rate was 68.044% indicated satisfactory construct validity.

**Figure 1:**
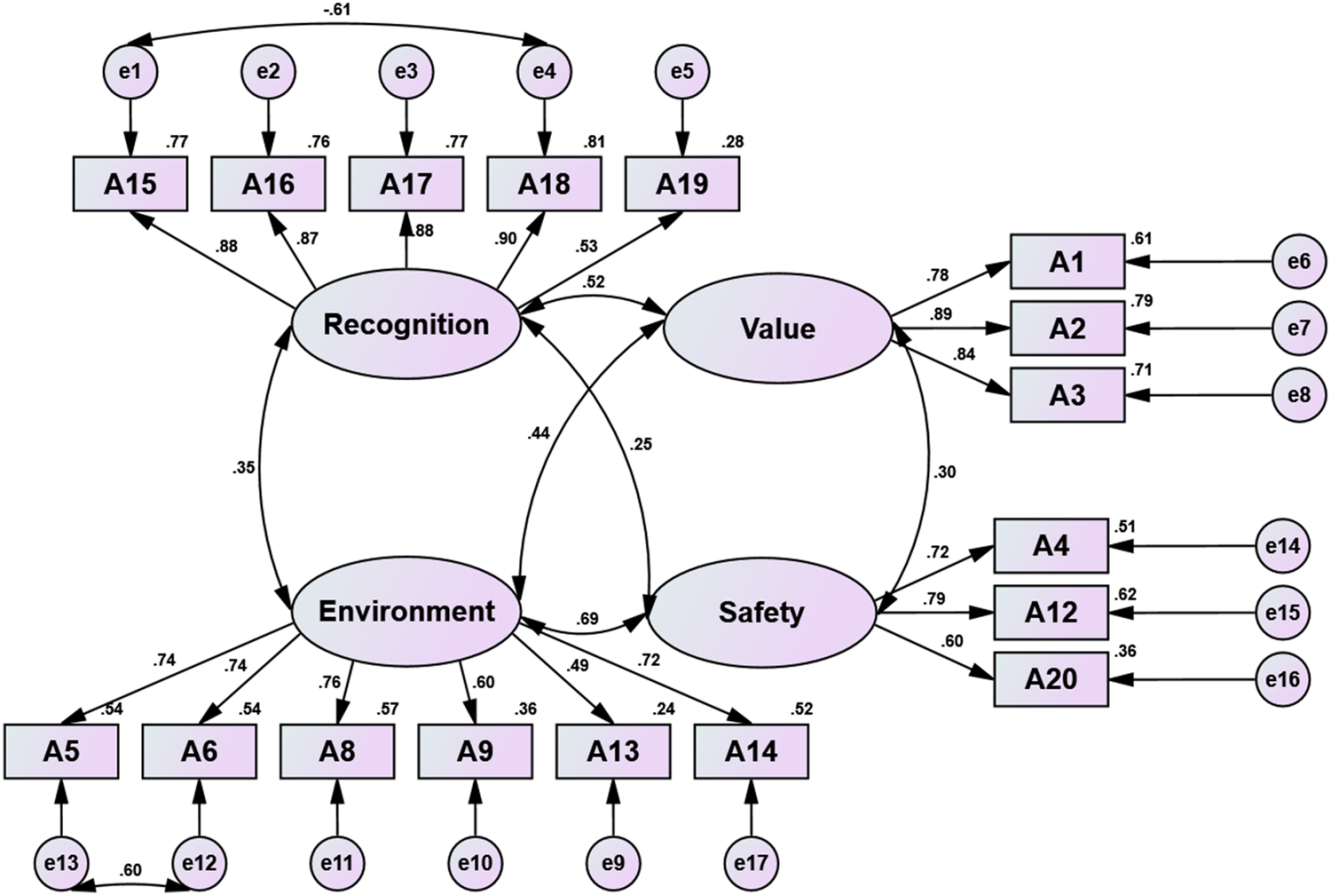
Confirmatory factor analysis of the acceptance attitudes towards vaccination scale.

A new four-factor structure was formed in Table 3. The Cronbach’s α of all factors was above 0.874, and the coefficients of recognition, environment, value and safety were 0.885, 0.839, 0.882 and 0.726, respectively. The CFA results were as follows: CMIN/df = 7.518, RMR = 0.042, GFI = 0.9530, AGFI = 0.0.935, NFI = 0.958, RFI = 0.949, IFI = 0.964, CFI = 0.964 and RMSEA = 0.056 (Fig. 1, Tables 4 and 5). All values of GFI, AGFI, NFI, RFI, IFI, TFI and CFI were above 0.9^30^. CMIN/df was greater than 3, RMSEA was less than 0.08, and RMR was less than 0.05, which indicated that the four-factor structure fit excellently^31^. When the sample size exceeds 1000, the chi-square value of the model will also increase, so CMIN/DF was not strictly considered^32^. All values of composite reliability (CR) of the four dimensions were greater than 0.7^33^, which means they achieved the required level. The values of average variance extracted (AVE) in Factors 2 and 4 were below but close to 0.5, indicating adequate construct reliability and adequate convergent validity^34^. For Factors 2 and 4, although the AVE value was less than 0.5, the other indicators were satisfactory; the CR values of the two factors were 0.835 and 0.747, respectively. Therefore, the factors were kept in the acceptance attitudes to vaccines scale. The square between the respective constructs was less than the square of AVE for the construct that achieved the discriminant validity of the acceptance attitudes towards vaccines.

**Table 3:** Factors and item loadings explored by exploratory factor analysis.

Factor number	Items included	Item loadings
1	A18 I would be more willing to get vaccinated if recommended by my family or friends	0.895
	A17 I would be more willing to get vaccinated if recommended by medical staff	0.891
	A16 I would be more willing to take vaccine if recommended by my workplace	0.880
	A15 I would be more willing to take vaccine if recommended by the country	0.832
	A19 Since the vaccine is free, I'm willing to take it	0.709
2	A6 Vaccination is not necessary for healthy people	0.921
	A5 If the infectious disease is effectively brought under control and vaccination is not necessary	0.894
	A14 I believe there are no new confirmed cases in the local area, so the vaccine is not required	0.805
	A8 I was hesitant about getting the coronavirus vaccine	0.704
	A9 I do not know much about vaccines	0.487
	A13 I think it is difficult to get the vaccine, especially during major infectious disease outbreaks (such as uncertain time and place of vaccination, inconvenient appointment, multiple vaccination, etc.)	0.430
3	A2 I think getting vaccinated reduces the likelihood of infection	0.930
	A3 I think vaccination against can reduce the rate of serious illness after infection	0.906
	A1 I think the whole population needs to be vaccinated	0.809
4	A 20 I would worry about the quality of vaccines made by different manufacturers	0.862
	A 12 I would worry about the protection of the vaccine	0.797
	A 4 I am concerned about the quality and safety of vaccines	0.682

**Table 4:** Convergent validity analysis table.

Factor no	Items	Factor loading	Reliability coefficient	Composite reliability	Average variance extracted
1	A15	0.880	0.774	0.910	0.678
	A16	0.870	0.757		
	A17	0.878	0.771		
	A18	0.898	0.806		
	A19	0.528	0.279		
2	A5	0.738	0.544	0.835	0.463
	A6	0.737	0.542		
	A8	0.756	0.571		
	A9	0.597	0.356		
	A13	0.491	0.241		
	A14	0.723	0.523		
3	A1	0.781	0.609	0.876	0.703
	A2	0.890	0.792		
	A3	0.840	0.705		
4	A4	0.716	0.513	0.747	0.498
	A12	0.789	0.622		
	A20	0.600	0.360

**Table 5:** Convergent validity and discriminant validity.

Factor number	AVE	1	2	3	4
1	0.678	0.823			
2	0.463	0.273	0.680		
3	0.703	0.485	0.380	0.838	
4	0.498	0.177	0.556	0.232	0.706

## Discussion

In this study, we created a scale for evaluating acceptance attitudes towards vaccination amidst a major public health crisis. A 17-item scale with four factors was confirmed: recognition (5 items), environment (6 items), value (3 items) and safety (3 items) formed the public acceptance attitudes towards vaccination scale.

Factor one was named ‘recognition’ and included 5 items (A15/A16/A17A18/A19). The Cronbach’s α value was 0.885, and item loading ranged from 0.528 to 0.898. They were all recommended and approved by authoritative institutions or trusted individuals, including the country, work units, medical staff, and family/friends. The price of vaccines plays a significant role in determining their affordability for individuals and serves as a primary factor in assessing vaccine acceptance. Recognition facilitates the rapid identification of factors influencing vaccine confidence and the establishment of manageable response strategies during public health emergencies. Confidence is a key influencing factor in vaccine hesitancy^35^, and recommendations from trusted individuals in the community can enhance individuals’ confidence in vaccine acceptance.

A research reveals that 87.09% of respondents deemed their doctor’s recommendation as crucial for decision-making^36^. Recognition also significantly influenced individuals' vaccination attitudes, serving as a cornerstone in fostering confidence and trust in the vaccination process. This contributed to increased vaccine acceptance amid major public health crises, as individuals prioritize safeguarding themselves and others from disease risks. However, the sudden and unpredictable nature of such crises can lead to fluctuations in vaccine acceptance. This is attributed to asymmetrical information regarding vaccines, where initial misinformation or incomplete knowledge may foster misunderstandings and resistance towards vaccination, fueling panic and distrust^37^. Yet, as validation of vaccine safety increases, public acceptance gradually improves. Therefore, recognition can swiftly identify controllable factors that foster positive attitudes towards vaccine acceptance, effectively mitigating the challenges posed by public crises.

Factor 2 was named ‘environment’ and included 6 items (A5/A6/A8/A9/A13/A14). The Cronbach’s α value was 0.839, and item loading ranged from 0.491 to 0.756. This factor represents the perception of the current risk of infectious disease outbreaks; if individuals perceive risk, they will behave to mitigate the risk. Vaccination is the most important response measure. Accordingly, people will exhibit positive response behaviour; otherwise, their behaviour involves hesitation and a lack of acceptance of the vaccine. A global survey indicates that individuals' attitudes towards vaccine acceptance have been influenced by the information disseminated through various social media platforms and the severity of COVID-19 cases^38^. Environmental experience is a crucial external factor determining individual vaccine acceptance^39^. According to surveys, there is a significant contrast in individuals’ attitudes towards vaccine acceptance during and after the COVID-19 pandemic^38,40^. When individuals are experiencing a major public health crisis like COVID-19, their perception of the imminent threat to life and health is deeper and more genuine, providing a more objective reflection of public attitudes towards vaccine acceptance or refusal. This differs from conventional factors influencing vaccine acceptance attitudes. However, after this period, vaccine acceptance attitudes are influenced by various sources of information, such as media reports and individual experiences. Therefore, the measurement of the environmental dimension in this study provides a more authentic and objective reflection of the current situation.

Factor 3 was named ‘value’ and included 3 items (A1/A2/A3). The Cronbach’s α value was 0.882, and item loading ranged from 0.781 to 0.8906. This factor captured opinions about the role and value of the vaccine. Whether an individual accepts a vaccine depends mainly on whether the vaccine can play a role in reducing infection. If the vaccine can reduce the infection rate and the rate of serious disease after infection, then the vaccine plays a role and can be positively recognized and accepted. The value dimension acts as a direct determinant of individual vaccine acceptance attitudes and also indirectly reflects individuals' health literacy regarding vaccines. Higher levels of health literacy regarding vaccines indicate a deeper understanding of the role and importance of vaccines in disease prevention. Conversely, lower levels of perceived value may signify knowledge gaps or misconceptions about vaccines, resulting in hesitancy or reluctance to accept vaccination. Understanding and addressing the factors influencing perceptions of vaccine value are essential for promoting vaccine acceptance and effectively addressing vaccine hesitancy^41^.

Factor 4 was named ‘safety’ and included 3 items (A4/A12/A20). The Cronbach’s α value was 0.726, and item loading ranged from 0.600 to 0.789. The three items were “quality and safety”, “protective effect”, “quality of the vaccine produced by the manufacturer” and all involved the theme of security. The most important consideration for an individual when getting vaccinated is the safety of the vaccine. The safety and efficacy of the vaccine are reasons for an individual to overcome hesitation, which is also in line with medical decision-making behaviour theory^42^. Safety is a key factor shaping individuals’ vaccine acceptance, especially during unforeseeable public health crises. When individuals are unsure about vaccine efficacy, safety becomes their primary concern^43^. Hence, strategies addressing safety concerns and providing transparent, accurate vaccine safety information are crucial for improving vaccination attitudes and uptake rates.

The scale is a unique and practical tool for assessing vaccination attitudes during a major public health crisis, providing distinct advantages and practical significance. Although a multitude of tools exist for assessing vaccine acceptance attitudes, including those tailored to COVID-19 vaccines, and extensive research has examined diverse predictive factors such as gender, age, education level, and economic development^35–41^, the emphasis of these investigations predominantly centers on uncontrollable variables. Addressing uncontrollable factors poses challenges for short-term resolution. During major public health crises, there is an urgent imperative to promptly tackle controllable factors that impact vaccine acceptance attitudes. The four dimensions of our research instrument are well-suited for evaluating vaccine acceptance attitudes amidst major public health crises. Recognition, environment, safety and value were the main factors in vaccine acceptance decision-making. Whether an individual makes a decision depends on both intrinsic and extrinsic factors centred on vaccine safety. Internal factors include vaccine safety and value, namely, whether the vaccine plays a reliable role. External factors include the urgency of the environment and the degree of recognition of the vaccine. Internal and external factors also involve weighing the pros and cons of vaccine acceptance or refusal. This can provide not only factors for public vaccine acceptance decision-making but also the weight of the factors to provide a basis for screening and decision-making for vaccine acceptance in different environmental settings.

The initial 5 items (A7/A10/A11/A21/A22/), which were about responses to individual knowledge of vaccines, were deleted and excluded from statistical analysis based on numerical exclusion criteria. In addition to statistical reasons, we analysed other possibilities. One was based on the realistic principle that individuals care more about the function and safety of vaccines than they do about the knowledge of vaccines. Previous studies have considered vaccine knowledge to be important^36^, but for people from non-medical backgrounds, especially in emergency settings, the safety and value of vaccines are considered more important. The other possibility is that individuals are very knowledgeable about vaccines because vaccines have been used successfully against many viruses throughout history. Finally, a statistical descriptive summary of several items showed that the scores ranged from 3.93 ± 0.88 to 4.66 ± 0.66, which were all relatively high. This indicated that knowledge of vaccines was not the main consideration for deciding whether to accept vaccines, but this specific explanation needs further research and analysis.

## Limitations

This study has several limitations. Firstly, the use of an online survey platform, while convenient and efficient, may introduce selection bias and raise concerns about response accuracy and misinterpretation of questions. Additionally, the inability to verify respondent identity online may compromise data validity. However, it’s worth noting that these limitations may not have significantly impacted the results. Secondly, the study exclusively utilized quantitative research methods to explore vaccination acceptance attitudes, suggesting a need for future research to incorporate qualitative approaches. Thirdly, retest reliability was not conducted, but it is planned for inclusion in a subsequent study. Lastly, the large sample size may potentially introduce ceiling or floor effects. To address this, we diversified sample sources and partitioned the dataset for exploratory and confirmatory factor analyses, aiming to mitigate these effects to some extent.

## Conclusions

We developed a scale for measuring acceptance attitudes towards vaccination and validated it with a relatively adequate sample size. This scale is a 17-item scale with 4 factors and excellent reliability and validity, making it acceptable and useful. It provides an effective evaluation tool for assessing whether individuals have adopted vaccination behaviour. The scale serves as a rapid assessment tool for identifying public vaccination acceptance attitudes, particularly during major public health crises. It enables healthcare practitioners to recognize and comprehend the key factors influencing vaccine acceptance attitudes, facilitating targeted health education and promotional campaigns aimed at altering public attitudes and behaviors, thereby promoting vaccination and reducing hesitancy. The dimensions assessed by the scale represent critical and controllable factors that the public primarily considers during major public health crises. Consequently, it can guide health policymakers in promptly understanding public attitudes toward vaccine acceptance and formulating more targeted and effective vaccination policies to enhance vaccination rates across diverse demographic groups. Additionally, healthcare professionals can employ the scale periodically to assess dynamic trends in vaccine acceptance attitudes and adjust relevant promotional and educational strategies in a timely manner, ensuring the effective implementation of vaccination activities. We look forward to witnessing the widespread application of this scale in practice and its contribution to addressing current and future global health challenges. Meanwhile, the scale can be used as a necessary part of the emergency plan system for screening and decision-making. The four dimensions in the scale contribute to building a knowledge system and model of vaccine acceptance, thereby enhancing strategies for major public health crisis prevention and control. Finally, this scale can be widely applied and tested in practice, contributing to addressing current and future global health challenges.

### Supplementary Information

Supplementary Information.

## Data Availability

The datasets used and/or analyzed during the current study are available from the corresponding author on reasonable request.

## Abbreviations

CMIN/df: Chi-square/df
RMR: Root mean square residual
GFI: Goodness-of-fit index
AGFI: Adjusted goodness-of-fit index
NFI: Normed fit index
RFI: Relative fit index
IFI: Incremental fit index
CFI: Comparative fit index
RMSEA: Root mean square error of approximation
VAX: Vaccination Attitudes Examination Scale
COVID-19: Coronavirus disease 2019
